# Proteasomal Processing Immune Escape Mechanisms in Platinum-Treated Advanced Bladder Cancer

**DOI:** 10.3390/genes13030422

**Published:** 2022-02-25

**Authors:** Michael Wessolly, Fabian D. Mairinger, Thomas Herold, Boris Hadaschik, Tibor Szarvas, Henning Reis

**Affiliations:** 1 Institute of Pathology, University Medicine Essen, University of Duisburg-Essen, 45147 Essen, Germany; michael.wessolly@uk-essen.de (M.W.); fabian.mairinger@uk-essen.de (F.D.M.); thomas.herold@uk-essen.de (T.H.); 2 Department of Urology, University Medicine Essen, University of Duisburg-Essen, 45147 Essen, Germany; boris.hadaschik@uk-essen.de (B.H.); tibor.szarvas@uk-essen.de (T.S.); 3 Department of Urology, Semmelweis University Budapest, 1085 Budapest, Hungary; 4 Dr. Senckenberg Institute of Pathology, University Hospital Frankfurt, Goethe University Frankfurt, 60596 Frankfurt, Germany

**Keywords:** bladder cancer, urothelial carcinoma, proteasomal processing escape, proteasome, platinum-based chemotherapy, immune therapy, immunohistochemistry

## Abstract

In recent years, the number and type of treatment options in advanced bladder cancer (BC) have been rapidly evolving. To select an effective therapy and spare unnecessary side effects, predictive biomarkers are urgently needed. As the host’s anti-cancer immune response is by far the most effective system to impede malignant tumor growth, immune system-based biomarkers are promising. We have recently described altered proteasomal epitope processing as an effective immune escape mechanism to impair cytotoxic T-cell activity. By altering the neoantigens’ characteristics through different proteasomal peptide cleavage induced by non-synonymous somatic mutations, the ability for T-cell activation was decreased (“processing escapes”). In the present study, we analyzed primary chemo-naïve tissue samples of 26 adjuvant platinum-treated urothelial BC patients using a targeted next-generation sequencing panel followed by the epitope determination of affected genes, a machine-learning based prediction of epitope processing and proteasomal cleavage and of HLA-affinity as well as immune activation. Immune infiltration (immunohistochemistries for CD8, granzyme B, CD45/LCA) was digitally quantified by a pathologist and clinico-pathological and survival data were collected. We detected 145 epitopes with characteristics of a processing escape associated with a higher number of CD8-positive but lower number of granzyme B-positive cells and no association with PD-L1-expression. In addition, a high prevalence of processing escapes was associated with unfavorable overall survival. Our data indicate the presence of processing escapes in advanced BC, potentially creating a tumor-promoting pro-inflammatory environment with lowered anti-cancerous activity and independence from PD-L1-expression. The data also need to be prospectively validated in BC treated with immune therapy.

## 1. Introduction

Bladder cancer (BC) is the ninth most common cancer worldwide with urothelial carcinoma comprising more than 90% of histologic subtypes [1,2]. Although 75–85% initially present as non-muscle invasive BC (NMIBC), survival rates are unfavorable at 50–60% in muscle-invasive BC (MIBC) and later stages [3]. In advanced disease, platinum-based combination chemotherapy has been the therapeutic gold standard for almost three decades [4]. However, approximately 50% of patients are ineligible for platinum-based treatment, which by itself is associated with serious toxicities [5]. In recent years, immune checkpoint blockade (ICB) has demonstrated effectiveness in cisplatin-ineligible patients and in progressive disease after chemotherapy [6]. In addition, ICB maintenance in patients that had not progressed with first-line chemotherapy demonstrated improved overall survival (OS) [7]. As ICB shows response rates of only 20–25% and further targeted therapy approaches emerge, identification of predictive biomarkers of therapeutic efficacy in advanced BC is of great clinical importance [8,9].

In chemotherapy, several predictive biomarkers have been reported [9]. For example, specific mutations in ERCC Excision Repair 2 (*ERCC2*) as well as alterations in other genes involved in DNA damage response and repair (DDR) sensitize to cisplatin efficacy in MIBC [10,11]. Further single predictive biomarkers have been reported [9,12] and the predictive value of molecular subtyping systems is a subject of current research [13,14].

Recently, the focus has also turned to the relevance of the immune system, which has also proven to be a key factor in cisplatin therapy efficacy [15,16]. From different tumor types, the role of tumor infiltrating lymphocytes (TILs) is well known. In this context, factors such as tumor neoantigens seem to play an important role in the tumor cell–immune cell interactions [17,18]. These tumor neoantigens pass through a complex intra-cellular mechanism before they can be effectively recognized by specific immune cells. The proteasome holds a key role in this process. A neoantigen destined for proteasomal degradation and fragment trimming gets polyubiquitinated and transferred into the proteasome in a linearized constitution. Depending on the chemical composition of its amino acids, it is processed to small peptide fragments and trimmed to an optimal length for the presentation of 8–11 amino acids. After transport into the endoplasmic reticulum (ER) via the transporter associated with antigen processing (TAP), the peptide fragments are loaded onto the HLA class I-complex. This complex is presented on the cell surface [17,18] and can be recognized by the T-cell receptor (TCR), depending on the neoantigen’s chemical properties in certain key positions. A failure of correct neoantigen processing might therefore result in failure of its TCR-recognition and thus in decreased anti-tumor immune activity.

We have previously shown that alterations in proteasomal antigen processing can be a general mechanism of immune escape in lung cancer [19]. In the present study, we analyzed if this mechanism is also effective in advanced platinum-treated BC.

## 2. Materials and Methods

### 2.1. Cohort

A single center cohort of 26 patients with advanced urothelial BC who underwent adjuvant platinum therapy and were resected at the Department of Urology at the University Hospital Essen was retrospectively constructed (1989–2010). Formalin-fixed and paraffin embedded (FFPE) tissues blocks were collected from the archive of the Institute of Pathology at the same institution. A genitourinary pathologist (HR) reviewed all cases. Clinico-pathological details are shown in Table 1 and Table S1.

### 2.2. Nucleic Acid Preparation

Genomic DNA was extracted using a semiautomatic DNA isolation kit (RSC DNA FFPE Plus Kit AX4920 custom, Promega Maxwell, Fitchburg, MA, USA) on a Maxwell RSC device (Maxwell RSC Instrument AS4500, Promega Maxwell) according to the manufacturer’s manual (R29X) with minor adaptions. Proteinase K end concentration was 20 mg/mL. Tumorous areas, identified by a board-certified pathologist, of two unstained 10 μm paraffin sections were used per case after macrodissection of marked tumorous areas (HR). The proteinase K mix was incubated over night at 70 °C (Eppendorf ThermoMixer F1.5, Eppendorf, Hamburg, Germany) and eluted in 70 µL nuclease free water (Plus Kit AX4920). DNA was quantified (Qubit1 fluorometer; Invitrogen, Carlsbad, CA, USA) using the Qubit dsDNA HS assay kit (Life Technologies, Gent, Belgium). DNA was diluted to 45 ng DNA/18 µL nuclease free water (Plus Kit AX4920) for a 4-pool panel.

### 2.3. Targeted Next-Generation Sequencing (NGS)

A small customized NGS panel was employed on purpose covering known cancer driver alterations (Table S2). Details have been described earlier [20]. In brief, after target enrichment, libraries were sequenced on an Illumina MiSeq platform (Illumina, San Diego, CA, USA) followed by mapping to the human genome (hg19). Variants were manually reviewed using the integrative genome viewer tool. Based on a tumor allelic frequency of 25% and above, 43 valid alterations were identified. Only non-synonymous mutations were included with relevance on protein level. To avoid bias, passenger mutations were also included in further analyses.

### 2.4. Immunohistochemistry (IHC)

**Figure 1:**
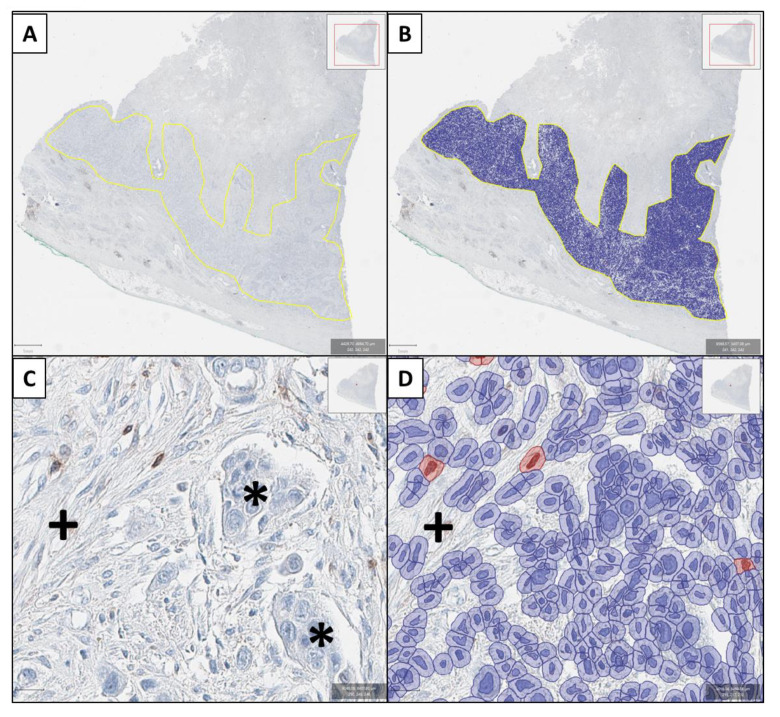
Example of the digital pathology approach (granzyme B immunostaining). In (**A**) the tumor area is identified by the genitourinary pathologist (HR) and after threshold adjustment, all cells are recognized (**B**). In (**C**) a tumor area showing cancer cells (*) and stromal cells (**+**) with few lymphocytes is shown. Only few lymphocytes show positive granzyme B-immunoreactivity with brown cytoplasmic staining and focal nuclear overlay. These cells are counted as positive (cells marked red in (**D**), while artificial brown background staining (**+**) is not recognized as a positive cell (**D**). Again, also all immunonegative cells are identified in (**D**) in hematoxylin staining.

Fresh serial 3 µm paraffin sections were cut from FFPE tumor blocks for IHC. An automized platform (Ventana Benchmark Ultra, Ventana medical systems, Oro Valley, AZ, USA) was used for immunostaining adherent to the manufacturer’s instructions. IHC slides were created using antibodies against CD8, granzyme B, leukocyte common antigen (LCA, CD45), and PD-L1 (Tables S3 and S4). Slides were scanned using an Aperio ScanScope AT2 platform (Leica biosystems, Wetzlar, Germany) and analyzed with QuPath (v.0.2.0-m2, qupath.github.io/; accessed on April 4 2019). A pathologist (HR) annotated all whole slides regarding the tumor area. After adjusting all images to DAB staining and the appropriate resolution, the number of positive cells within the tumor area was counted. A pathologist (HR) verified all steps of the image analysis and threshold adjustments for correct identification of positive and negative cells and artifacts (Figure 1). For PD-L1 analyses, the PD-L1 combined positive score (CPS) and the immune cell score (IC-score) were evaluated in every case [21].

### 2.5. Statistical and Bioinformatical Analysis

All bioinformatical, statistical, and graphical analyses were performed using the R programming environment.

#### 2.5.1. Epitope Search of Affected Genes

For every non-synonymous mutation in a cancer-related gene, epitope databases (e.g., Immune Epitope Database, IEDB) were browsed [22]. If a mutation was associated with an epitope (length: nine amino acids), all relevant data were extracted, including the amino acid sequence and the HLA molecule with the highest binding affinity. The epitope sequences were used as input for proteasomal cleavage prediction.

#### 2.5.2. Prediction of Epitope Processing and Proteasomal Cleavage

NetChop 3.1 is a machine-learning algorithm based on convolutional neural networks. It was trained to recognize cleavage positions given a specific amino acid sequence [23,24]. Two different network methods, NetChop 20S and NetChop Cterm, were available. NetChop 20S was trained on in vitro generated data for proteasomal digestion [25] while NetChop Cterm was trained to recognize the structure of MHC class I molecules based on in vivo data [26]. NetChop Cterm was chosen as the primary network method. Before epitope sequences were submitted to NetChop, it was necessary to consider the chemical structure of amino acids flanking the epitope, thereby also influencing proteasomal cleavage. In the literature, the impact of flanking regions onto the cleavage of epitope start and end has been reported both experimentally and in silico. For example, mutations in HIV genes outside the actual epitope were reported to play a major role in epitope-processing based immune impairment and the resulting cleavage pattern. All relevant mutations were located within a maximum distance to the epitope borders of eight amino acids. Nevertheless, most occur within a range of a maximum of five amino acids outside the epitope [27,28,29]. To ascertain the most information from our data, we decided to submit sequences of 25 amino acids (eight in the N- and C-terminal flanking regions, respectively, and nine in the epitope region). NetChop outputted a cleavage probability for every amino acid position. For control purposes, a wild-type epitope sequence, excluding the non-synonymous mutation, was also analyzed in each case. If the cleavage probability for a specific position differed more than 50%, the mutation was considered to alter the proteasomal processing of an epitope. The 50% cutoff value was chosen, as in the concept of direct selection pressure. As a basis of mutation selection, the resulting epitope variant has to be a predominant one, as T cell priming is based on the composition of cell surface proteins rather than on single epitopes in minor numbers.

#### 2.5.3. In Silico Prediction of HLA-Affinity and Immune Activation

In order to verify the MHC binding capability of altered epitopes, NetMHC 4.0, another machine-learning algorithm, was utilized [30,31]. NetMHC 4.0 predicts the binding affinity of a given epitope to a specific HLA molecule. To cover a wide range of potential binding partners, the binding affinity of epitopes towards 12 HLA supertypes was calculated [32,33,34,35]. The final output contained the binding affinity (IC50) for each respective HLA supertype as well as the general binder status of an epitope (binders/non-binders).

The potential of an epitope to trigger TCR activation of a cytotoxic lymphocyte was estimated using the Class I Immunogenicity tool [36].

#### 2.5.4. Statistical Analysis in R

Statistical tests for each possible correlation were calculated based on the type of variable and group size. In case a metric variable was compared to a categorical variable, the metric variable was subjected to a Shapiro–Wilk test. Depending on the data distribution, a parametrical (Student’s *t*-test) or a non-parametrical test (Wilcoxon rank-sum test) was applied. For the comparison of two categorical variables containing two groups each, a double dichotomous contingency table was generated. The dependence of both variables was calculated by Fisher’s exact test. If one categorical variable was comprised of more than two groups, the Pearson’s chi-squared test was utilized instead. In order to compare the dependency of two metric variables, Pearson’s product moment correlation coefficient (linear modeling) and Spearman´s rank correlation test (non-linear modeling) were applied. To test the influence of a specific variable on patients’ survival (OS) and/or progression-free survival (PFS), a Cox proportional hazard model was designed. A *p*-value estimated by Wald-test, likelihood-ratio test, and Score (log-rank) test served as an indication for the significant impact a certain variable has on OS/PFS. In order to address the multiple comparison problems when calculating the *p*-value, they were corrected utilizing the false discovery rate (FDR). The level of statistical significance was defined as *p* ≤ 0.05 after adjustment.

## 3. Results

Detailed clinico-pathological, molecular, and immunohistochemical data of the cohort are shown in Table S1.

### 3.1. Processing Escapes in Bladder Cancer

A total of 43 non-synonymous variants were identified. Of these, 51% (*n* = 22) were associated with altered epitope processing. The number of epitopes affected by each single mutation ranged from one to eight per non-synonymous mutation (median: *n* = 1), resulting in 250 predicted neoantigens. Of those, 121 (48%) were no longer considered as ligands for the MHC class I complex as the resultant epitope lengths were outside the range of possible transport and presentation capabilities. Another 60 (24%) epitopes were predicted to bind neither in their mutated nor in their wild-type state, which results in 69 (28% of the initially *n* = 250) epitopes predicted to be present on the MHC class I complex. Of those 69 epitopes, 29 (42%) were predicted to preferably bind in their mutated state and of these 29 epitopes, five (17%) were calculated to have the ability to activate the immune system. The remaining 40 epitopes have been identified as non-binders, losing their initial affinity for all HLA supertypes.

### 3.2. Processing Escapes Do Not Correlate with PD-L1 Status

**Figure 2:**
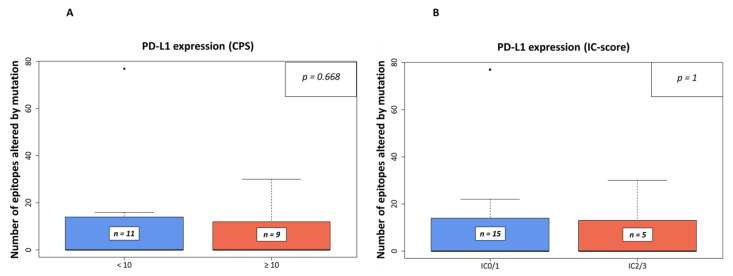
Number of epitopes altered by mutation in relation to PD-L1 expression in IHC-analyses. In (**A**), the relation is shown to PD-L1 expression using the combined positive score (CPS) with a threshold of 10 as used in companion diagnostic PD-L1-IHC analyses for pembrolizumab in the first-line setting of urothelial BC, while in (**B**), the threshold was set to an IC-Score of ≥ 5% (IC0/1 vs. IC2/3) as used in the same setting for atezolizumab [21]).

The number of affected epitopes was analyzed in relation to the PD-L1 CPS (Figure 2A) and the IC-scores (Figure 2B). No significant association between processing escapes and both scores (*p* = 0.668, *p* = 1, respectively) was noted.

### 3.3. Processing Escapes and Immune Infiltrate Characteristics

Spearman’s rank correlation tests were performed to analyze associations between the number of affected epitopes and specific immune cell counts (represented by cells positively stained for CD8, granzyme B, or LCA) represented by quantified IHC-staining results (Table S4). The cell count of immune cells was dichotomized according to their median level (Tables S5 and S6). In addition, immune cell counts were normalized to the total number of infiltrating leucocytes in LCA immunostaining. No significant associations between the amount of processing escapes and specific immune cell marker counts were detected (all: *p* > 0.05).

**Figure 3:**
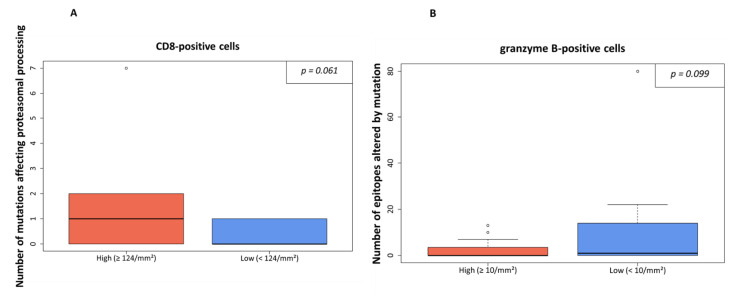
Processing escapes and CD8-/granzyme B-positive cell rate. In (**A**), the infiltration of CD8-positive cells is shown in relation to numbers of non-synonymous mutations influencing proteasomal processing. The amount infiltrating lymphocytes was either high (≥124/mm^2^) or low (cutoff level: median value). In (**B**), the number of granzyme B-positive cells is shown in relation to the numbers of epitopes altered by mutation. The threshold was set at ≥10/mm^2^ as indicated by the median value.

However, when dichotomized at median level, a trend to a positive association of a higher rate of processing mutations and higher number of CD8-positive cells was detected (*p* = 0.061, Figure 3A, cutoff level: 124 CD8-positive cells per mm^2^). An opposite trend was detected for granzyme B positive cells, which were higher in cases with a lower number of affected epitopes and vice versa (*p* = 0.099; Figure 3B, cutoff level: 10 granzyme B-positive cells per mm^2^).

**Figure 4:**
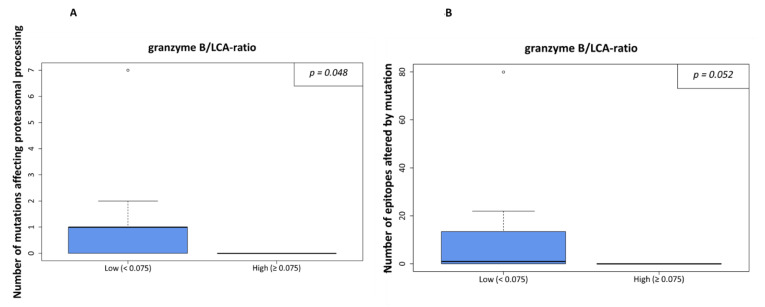
Processing escapes and proportion of granzyme B-positive cells from all leucocytes (LCA-positive). In (**A**), the ratio is shown in relation to numbers of non-synonymous mutations influencing proteasomal processing. The cutoff was set at 7.5% as density distributions exhibited a bimodal distribution with an optimal threshold value at 0.075. In (**B**), the same calculation is shown for the number of epitopes altered by mutation.

The latter was also true in the case of the ratio of granzyme B- of all LCA-positive immune cells. In cases of a higher rate of mutations affecting proteasomal processing or a higher rate of epitopes affected by mutation, lower granzyme B/LCA-ratios were detected (*p* = 0.048, Figure 4A, and *p* = 0.052, Figure 4B, respectively). However, the ratio of granzyme B positive cells to CD8 positive cells was not significantly different regarding the amount of processing mutations (*p* = 0.534) or number of epitopes affected by mutations (*p* = 0.941).

### 3.4. Patients with a High Number of Epitopes Affected by Mutation Have Unfavorable OS and PFS

**Figure 5:**
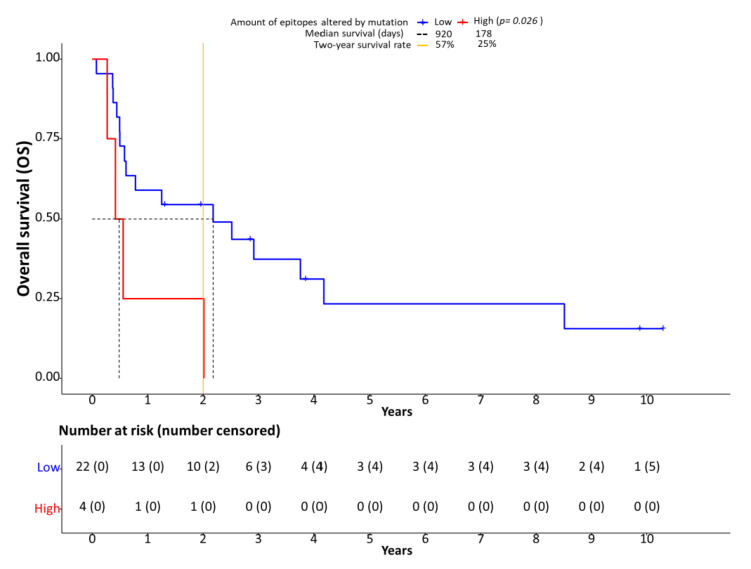
Processing escapes and survival. Kaplan-Meier plots showing overall survival (OS) in relation to the number of epitopes altered by mutation. The cutoff between low and high number of epitopes altered by mutation was set at 20 based on bimodal value distribution.

Patients were separated in two groups based on low or high numbers of epitopes altered by mutations. The number of affected epitopes is bimodally distributed (Figure S1), allowing categorization into a low (*n* = 22) and high number of epitopes (*n* = 4) group. The most accurate separation of both groups was achieved at 20 affected epitopes. Survival analyses showed significantly shortened OS (HR: 1.80, 95% CI: 0.11–3.50, *p* = 0.026, Figure 5) and PFS (HR: 1.70, 95% CI: 0.04–3.35, *p* = 0.034) for patients with 20 or more epitopes altered by mutations associated with altered proteasomal processing. However, we did not detect a statistically significant correlation of the number of CD8-positive cells and survival.

## 4. Discussion

Cancer cells, including urothelial bladder carcinoma cells, are under constant selective pressure from various factors. In this scenario, the T-cell based immune reaction is an effective anti-cancer system that needs to be evaded by cancer cells in order to survive. A critical point of the T cell–cancer cell interaction is the ability to identify tumor cells by cell surface autoantigens, which is established by the T-cell-receptor (TCR) interaction with MHC class I bound neoantigens of the cancer cells. After its recognition, the neoantigen-harboring cell gets attacked by cytotoxic T-cells (CD8+) through secreted granzymes (for example granzyme B) and perforin [37]). The anti-cancer effectiveness of cytotoxic T-cell is increased by the higher number and recognizability of the aberrant antigens presented on the cancer cell surface caused by somatic mutations. Therefore, cancer cells need to evade the immune response, e.g., by employing the PD-1/PD-L1 immune checkpoint to impair T-cell mediated tumor cell destruction [38,39]. In addition, altering the neoantigen presentation provides the cancer cell another way of hindering effective T-cell response. One way of achieving this is altering the presented protein fragment’s size and therefore affinity to bind to the MHC-class I-complex or recognizability to the TCR, which has been identified as a pivotal mechanism in viral infections [40,41]. We have recently shown this mechanism to be active through exonic mutations leading to the altered proteasomal processing of epitopes (“processing escapes”) in lung cancer [19]. As a result of changes in the chemical composition of the amino acid sequence due to non-synonymous somatic mutations, the proteasomal cleavage properties may change as the different proteasomal subunits show different cleavage preferences for acidic, basic, or hydrophobic amino acids [42]. In addition, the immune proteasome, which is induced by interferon γ secretion during an active immune response, further specializes towards the cleavage of hydrophobic sidechains [42,43,44]. With cleavage preferences modified by mutations, differential epitope variants can occur with variations in length from wild-type epitopes [40,41]. This can lead to a loss of the neoantigen’s binding affinity to the MHC class I-complex or altered binding affinity of the complex to the TCR, finally resulting in non-effective cytotoxic T-cell activation. In the present study, we aimed to analyze the mechanism in advanced urothelial BC.

To create a real-world scenario, we used genomic data obtained intentionally from a small targeted next generation sequencing panel covering 17 known cancer driver genes (Table S2). No additional new or unknown DNA-regions were analyzed making this approach interesting for potential inclusion in routine diagnostics. In addition, we chose a cohort of chemo-naïve BC with postoperative platinum-containing chemotherapy as it is known that platinum-containing chemotherapy at least moderately increases neoantigen burden [45]. We theorize that this effect might increase the processing escapes’ impact. However, although the adjuvant regimens used here all contained platinum (Table S1), additional agents, such as gemcitabine as a DNA intercalator or methotrexate, might have influenced the results. Due to the small group size, however, these effects could not be analyzed in cohort.

In our digital quantification approach, we indeed detected an effect on the immune infiltrate in the presence of processing escapes. A higher number of CD8-positive (cytotoxic) cells (Figure S2) was present in the tumor area when more mutations affecting proteasomal processing were predicted (Figure 3A). Although the statistical significance level was missed in our small sample (*p* = 0.061), it seems that more cytotoxic cells are attracted to the tumor area. However, as in parallel the rate of granzyme B positive cells tended to be lower in the case of more epitopes being affected by mutation (*p* = 0.099, Figure 3B), the anti-cancer effect of the increased CD8-positive cell infiltration seems to be ineffective. This notion is supported by the negative prognostic impact of the presence of processing escapes both on PFS and OS (Figure 5). As the presence of processing escapes was independent from the PD-L1 expression status measured both on the tumor and immune cell level by calculation of the CPS and IC-scores (Figure 2), one can theorize that the analyzed mechanism in this study is another type of immune evasion that cancer cells can employ.

However, the observed higher rate of CD8-positive cytotoxic T-cells in the presence of processing escapes remains counter-intuitive and no association with OS or PFS was noted, potentially due to the small sample size. It is possible that the tumor attracts immune cells for its own purposes, while simultaneously evading the anti-tumor response. It has been demonstrated that tumor-promoting effects can be induced by an inflammatory process at the tumor site, especially by cells of the innate immune response [46,47,48].

Our study has to be considered as a proof-of-concept study that has some limitations. As we analyzed CD8 and granzyme B by IHC, we cannot exclude the potential detection of some NK cells. We therefore chose a general terminology. We also analyzed a small cohort of retrospectively collected samples, which reduces the statistical power despite our efforts to correct for overfitting errors. In addition, the computed presence or absence of processing escapes cannot be proven by another method. However, as the used algorithms are developed and validated in the context of large external projects and we detected a prognostic influence, the obtained data seem to hold biological significance. In addition, we did not analyze samples with ICB-therapy, which would be of significant interest for further studies.

## 5. Conclusions

In conclusion, to the best of our knowledge, we present the first data suggesting a potential immune escape mechanism by altered proteasomal antigen processing (“proteasomal processing escapes”) in advanced urothelial BC. The mechanism seems to be active in this cancer type holding both functional effect, as shown by altered immune infiltration, and prognostic value. As this study was based on the analysis of a small retrospective cohort, further studies are needed to validate our findings. In addition, a prospective study analyzing the effects in advanced BC treated with immune therapy would potentially allow the analysis of the mechanism’s therapy predictive value, which would be of great interest.

## Figures and Tables

**Table 1:** Clinico-pathological data of the cohort.

		*n* (%)
Sex	female	4 (15)
	male	22 (85)
Age (mean, y)	61.4	
Stage (pT)	1	0 (0)
	2	4 (15)
	3	16 (62)
	4	6 (23)
LN (pN)	0	11 (42)
	1	15 (58)
Metastasis (M)	0	15 (58)
	1	11 (42)
Grade	1 (LG)	0 (0)
	2 (HG)	5 (19)
	3 (HG)	21 (81)
Adj. treatment	Gem/Cis	18 (69)
	MVEC	4 (15)
	Cis/Carbo/MTX	1 (4)
	Cis/MTX	3 (12)
Follow-up	dead	20 (77)
	alive	6 (23)
OS	mean (mo)	29
	median (mo)	15.3
PFS	mean (mo)	24.8
	median (mo)	9

All cases were classified according to the 7th edition of the TNM Classification of Malignant Tumors. Y: years, LN: lymph node, LG: low grade, HG: high grade, Gem/Cis: Gemcitabin/Cisplatin, MVEC: Methotrexate/Vinblastine/Epirubicin/Cisplatin, Cis/Carbo/MTX: Cisplatin/Carboplatin/Methotrexate, Cis/MTX: Cisplatin/Methotrexate, OS: overall survival, mo: months, PFS: progression free survival.

## Data Availability

All curated sequencing data obtained in this study is included in the Supplementary Material. Additional raw data including information on processing escape mechanism calculation can be obtained from the first authors (M.W.).

## Supplementary Materials

The following are available online at https://www.mdpi.com/article/10.3390/genes13030422/s1, Figure S1: The number of affected epitopes is bimodally distributed; Figure S2: Microphotographs of the immunostainigs; Table S1: Detailed clinico-pathological, molecular and immunohistochemical data of the cohort; Table S2: Covered exonic regions in targeted next generation sequencing analyses; Table S3: Detailed antibody protocol information for immunohistochemistry; Table S4: Supplementary contingency table of all immunohistochemical markers; Table S5: metric correlations for each immune marker, Table S6: dichotomous correlations for each immune marker.
