# Supplementary material for: Proteasomal Processing Immune Escape Mechanisms in Platinum-Treated Advanced Bladder Cancer
Source: Genes (Basel). 2022 Feb 25;13(3):422. doi: 10.3390/genes13030422 (PMC8948673; doi:10.3390/genes13030422)
Supplement: Supplementary file 1 [file genes-13-00422-s001.zip › FigureS1_R2.pdf]

Figure S1

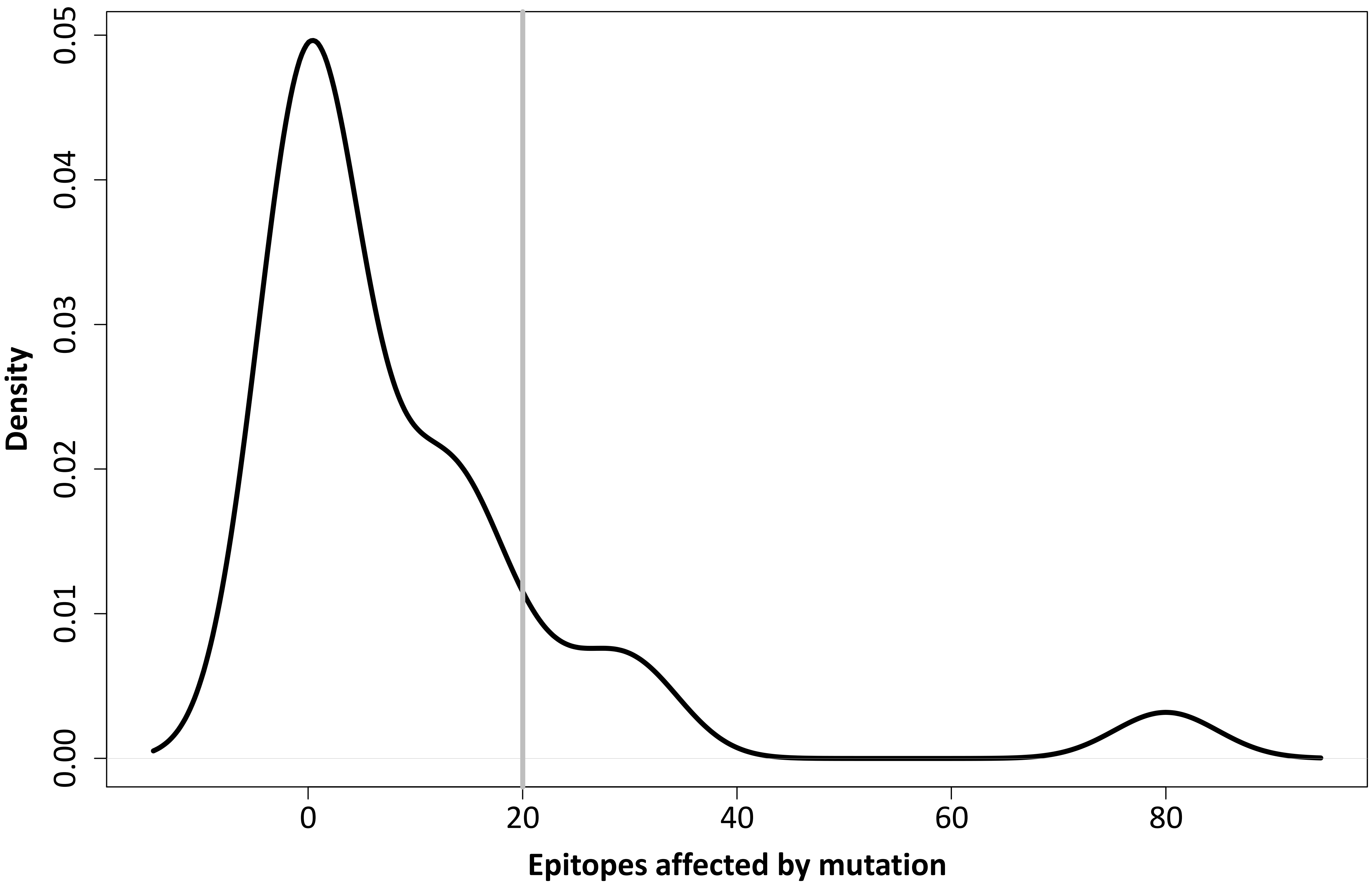

Figure S1: Density distribution of the affected epitopes.
This plot visualizes the density or amount of data points (y-axis) around discrete epitope frequencies (x-axis).
The shape of the density curve highlights a three distinct clusters (Around 0, 20 and 80 epitopes). The second cluster, that is distinct from the 0 forms around an epitope frequency of 20. Therefore, we chose 20 as our threshold to distinguish low (n = 22) and high amount of epitopes (n=4).
