# Supplementary material for: Proteasomal Processing Immune Escape Mechanisms in Platinum-Treated Advanced Bladder Cancer
Source: Genes (Basel). 2022 Feb 25;13(3):422. doi: 10.3390/genes13030422 (PMC8948673; doi:10.3390/genes13030422)
Supplement: Supplementary file 1 [file genes-13-00422-s001.zip › FigureS2 R2.pdf]

Figure S2

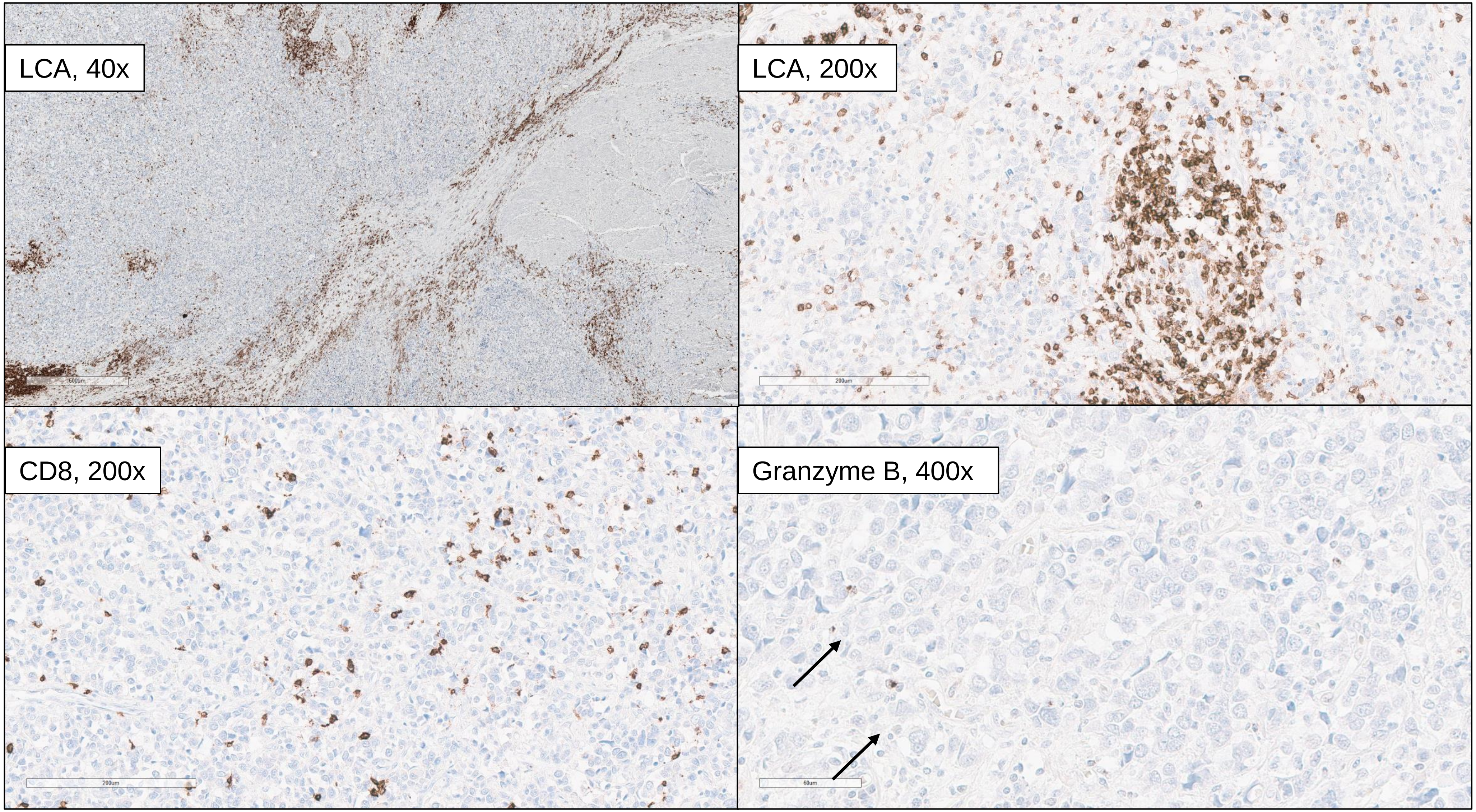

Figure S2: Microphotographs of the immunostainings.

Top row: LCA (leucocyte common antigen (CD45) in 40x and 200x magnification. Immune cells are accentuated at the tumor periphery, but also in the tumor itself; CD8-positive cells are less often detected and for the detection of granzyme B positive cells, the algorithm had to be adjusted due to small granular cell positivity. This needs a well performed immunohistochemical process, as artifacts would have hindered correct detection.
