# Supplementary material for: Proteasomal Processing Immune Escape Mechanisms in Platinum-Treated Advanced Bladder Cancer
Source: Genes (Basel). 2022 Feb 25;13(3):422. doi: 10.3390/genes13030422 (PMC8948673; doi:10.3390/genes13030422)
Supplement: Supplementary file 1 [file genes-13-00422-s001.zip › TableS1.pdf]

Table S1. Detailed clinico-pathological, molecular and immunohistochemical data of the cohort

Clinico-pathological data													Molecular data						IHC data (PD-L1)		
Id	Sex	Age at diagnosis (y)	Tumor stage (pT)	LN stage (pN)	Metastasis (M)	Grade	UC subtype (yes/no)	UC subytpe	Treatment	OS (days)	Status	PFS (days)	Gene	Alteration	Gene	Alteration	Gene	Alteration	PD-L1 TPS (%)	PD-L1 CPS	PD-L1 IC-Score (%)
UC_01	male	49	4	1	1	3	no	100% NOS	MVEC	3107	dead	3107	HRAS	c.34G>A	TP53	c.715G>A			5	6	1
UC_02	male	66	4	0	1	3	n/a	n/a	Cis/MTX	138	dead	55	TP53	c.339C>G							
UC_03	male	51	3	1	0	3	no	100% NOS	Gem/Cis	478	alive	478	TP53	c.991C>T					15	80	50
UC_04	male	51	4	1	1	3	no	100% NOS	Cis/Carbo/MTX	285	dead	231	TP53	c.978delA					20	25	4
UC_05	female	54	3	1	0	3	yes	100% plasmacytoid	Gem/Cis	162	dead	162	TP53	c.659A>G					70	72	1
UC_06	male	64	2	0	1	2	n/a	n/a	Cis/MTX	204	dead	125	TP53	c.839G>A	TP53	c.538G>A					
UC_07	male	69	3	1	0	3	yes	95% NOS, 5% MPUC	Gem/Cis	1041	alive	317	TP53	c.617T>A					0	3	2
UC_08	male	48	3	0	0	3	n/a	n/a	Gem/Cis	28	dead	28	TP53	c.637C>T							
UC_09	female	51	2	1	0	2	no	100% NOS	Gem/Cis	736	dead	622	FGFR3	c.1118A>G	PIK3CA	c.1048G>A	PIK3CA	c.3140A>G	80	90	15
UC_10	female	83	3	1	0	3	yes	80% NOS, 20% MPUC	Gem/Cis	181	dead	181	KRAS	c.34G>T					8	8	0
UC_11	male	63	3	0	1	3	yes	80% NOS, 20% glandular	Gem/Cis	1406	alive	211	TP53	c.743G>A					75	85	7
UC_12	female	68	2	1	0	2	yes	100% MPUC	Gem/Cis	716	alive	716	wt						30	30	0
UC_13	female	39	3	1	0	2	yes	80% squamous, 20% NOS	Gem/Cis	135	dead	23	PIK3CA	c.1633G>A	TP53	c.890_900del11			3	8	6
UC_14	male	77	3	1	1	3	n/a	n/a	Gem/Cis	1522	dead	1522	TP53	c.839G>C							
UC_15	male	74	3	0	1	3	yes	100% sarcomatoid	Gem/Cis	99	dead	99	TP53	c.374_375delCGinsTT	TP53	c.743G>A	HRAS	c.34G>A	60	60	0
UC_16	male	68	2	1	0	2	no	100% NOS	Gem/Cis	213	dead	191	wt						0	0	0
UC_17	male	70	3	0	1	3	no	100% NOS	Gem/Cis	920	dead	556	FGFR3	c.742C>T	TP53	c.853G>T			0	1	1
UC_18	male	55	3	1	1	3	no	100% NOS	Gem/Cis	223	dead	136	TP53	c.743G>A					60	65	3
UC_19	male	60	4	0	0	3	yes	100% plasmacytoid	Gem/Cis	1372	dead	1277	wt						3	3	6
UC_20	male	69	4	0	0	3	no	100% NOS	MVEC	153	dead	19	STK11	c.971C>T	ERBB2	c.3235G>A			0	2	2
UC_21	male	41	3	0	0	3	n/a	n/a	Gem/Cis	797	dead	797	wt								
UC_22	male	65	3	0	0	3	no	100% NOS	MVEC	3604	alive	3604	PIK3CA	c.1624G>A	TP53	c.223C>T	STK11	c.408G>A	2	4	1
UC_23	male	61	3	1	0	3	no	100% NOS	MVEC	3757	alive	3757	KRAS	c.35G>A					2	3	2
UC_24	male	59	4	1	1	3	no	100% NOS	Cis/MTX	458	dead	336	TP53	c.488A>G					0	1	1
UC_25	male	70	3	1	0	3	n/a	n/a	Gem/Cis	1065	dead	1065	TP53	c.833C>G	STK11	c.971C>T					
UC_26	male	75	3	0	1	3	yes	80% NOS, 20% MPUC	Gem/Cis	182	dead	72	TP53	c.839G>A	TP53	c.538G>A			20	25	3

All cases were classified according to the 7th edition of the TNM Classification of Malignant Tumors
IHC: immunohistochemistry, Id: identification number, Y: years, LN: lymph node, UC: urothelial carcinoma, OS: overall survival, PFS: progression free survival, PD-L1 TPS: PD-L1 tumor proportion score, PD-L1 CPS: PD-L1 combined positive score, PD-L1 IC-Score: PD-L1 immune cell score, n/a: data not available, NOS: not otherwise specified, MPUC: micropapillary urothelial carcinoma, MVEC: Methotrexate/Vinblastine/Epirubicin/Cisplatin, Cis/MTX: Cisplatin/Methotrexate, Gem/Cis: Gemcitabin/Cisplatin, Cis/Carbo/MTX: Cisplatin/Carboplatin/Methotrexate, mo: months, wt: wild-type.
