# Supplementary material for: Proteasomal Processing Immune Escape Mechanisms in Platinum-Treated Advanced Bladder Cancer
Source: Genes (Basel). 2022 Feb 25;13(3):422. doi: 10.3390/genes13030422 (PMC8948673; doi:10.3390/genes13030422)
Supplement: Supplementary file 1 [file genes-13-00422-s001.zip › TableS2.pdf]

Table S2. Covered exonic regions in targeted next generation sequencing analyses

Gene	covered exons
BRAF	11,15
EGFR	18-21
ERBB2	5,6,15,20,23,29
FGFR1	3,7,13,17
FGFR3	7,9
HRAS	2-4
IDH1	4
IDH2	4
KIT	9,10,11,13,17,18
KRAS	2-4
MET	3,8,11,14,19
NRAS	2-4
PDGFRa	12,14,18
PIK3CA	3,5,10,16,21
RET	7,10,11,13-16
STK11	1-9
TP53	2-11
