# Supplementary material for: Proteasomal Processing Immune Escape Mechanisms in Platinum-Treated Advanced Bladder Cancer
Source: Genes (Basel). 2022 Feb 25;13(3):422. doi: 10.3390/genes13030422 (PMC8948673; doi:10.3390/genes13030422)
Supplement: Supplementary file 1 [file genes-13-00422-s001.zip › TableS3.pdf]

Table S3. Detailed antibody protocol information for immunohistochemistry

Antibody	Vendor; Clone / #	Pretreatment	Dilution	Incubation	Detection system
PD-L1	Abcam; 28-8	CC1, 90°C, 40min	1:250	36°C, 32min	OptiView DAB IHC Detection Kit
LCA (CD45)	Dako; 2B11 / PD7/26	CC1, 90°C, 32min	1:1000	36°C, 24min	OptiView DAB IHC Detection Kit
CD8	Dako; C8/144B	CC1, 95°C, 40min	1:150	36°C, 24min	OptiView DAB IHC Detection Kit
Granzyme B	Novocastra; 11F1	CC1, 90°C, 32min	1:80	36°C, 32min	OptiView DAB IHC Detection Kit
