# Supplementary material for: Proteasomal Processing Immune Escape Mechanisms in Platinum-Treated Advanced Bladder Cancer
Source: Genes (Basel). 2022 Feb 25;13(3):422. doi: 10.3390/genes13030422 (PMC8948673; doi:10.3390/genes13030422)
Supplement: Supplementary file 1 [file genes-13-00422-s001.zip › TableS4 R2.pdf]

Table S4: Supplementary contingency table of all immunohistochemical markers

	Q1	Q2	Q3	Q4
**GZMB: Positive cell detection per mm² (Q0/1/2/3/4: 1.279/4.31225/8.836/17.4475/241.57)**	6	6	6	6
**LCA: Positive cell detection per mm² (Q0/1/2/3/4: 29.18/175.81/323.115/700.675/1948.8)**	6	6	6	6
**CD8: Positive cell detection per mm² (Q0/1/2/3/4: 0.6792/52.01/124.84/385.03/3141)**	7	6	6	6
**PD-L1 / TPS: H-Score (Q0/1/2/3/4: 0/1.5/6.5/37.5/80)**	5	5	5	5
**PD-L1 / CPS: H-Score (Q0/1/2/3/4: 0/3/8/61.25/90)**	7	4	4	5
**PD-L1 / IC-Score: H-Score (Q0/1/2/3/4: 0/1/2/4.5/50)**	9	3	3	5

Supplementary Table S4: contingency table of all immunohistochemical markers

GRZMB: granzyme B, LCA: leucocyte common antigen (CD45), PD-L1: Programmed death-ligand 1, TPS: Tumor proportion score, CPS: combined positive score, IC-Score: Immune cell score.
