# Supplementary material for: Proteasomal Processing Immune Escape Mechanisms in Platinum-Treated Advanced Bladder Cancer
Source: Genes (Basel). 2022 Feb 25;13(3):422. doi: 10.3390/genes13030422 (PMC8948673; doi:10.3390/genes13030422)
Supplement: Supplementary file 1 [file genes-13-00422-s001.zip › TableS5 R2.pdf]

Table S5: metric correlations for each immune marker.

variable 1	variable 2	test	normalization	p-value
Number of affected epitopes	GZMB	Spearman's Rank Correlation Test	None	0.9175
Number of affected epitopes	LCA	Spearman's Rank Correlation Test	None	0.9318
Number of affected epitopes	CD8	Spearman's Rank Correlation Test	None	0.4612
Number of affected epitopes	GZMB normalized	Spearman's Rank Correlation Test	To the total number of leucocytes (LCA)	0.6128
Number of affected epitopes	CD8 normalized	Spearman's Rank Correlation Test	To the total number of leucocytes (LCA)	0.7608
Number of affected epitopes	PD-L1 TPS	Spearman's Rank Correlation Test	None	0.5471
Number of affected epitopes	PD-L1 CPS	Spearman's Rank Correlation Test	None	0.5989
Number of affected epitopes	PD-L1 IC-Score	Spearman's Rank Correlation Test	None	0.9096

Supplementary Table S5: metric correlations for each immune marker.

GRZMB: granzyme B, LCA: leucocyte common antigen (CD45), PD-L1: Programmed death-ligand 1, TPS: Tumor proportion score, CPS: combined positive score, IC-Score: Immune cell score.
