# Supplementary material for: Proteasomal Processing Immune Escape Mechanisms in Platinum-Treated Advanced Bladder Cancer
Source: Genes (Basel). 2022 Feb 25;13(3):422. doi: 10.3390/genes13030422 (PMC8948673; doi:10.3390/genes13030422)
Supplement: Supplementary file 1 [file genes-13-00422-s001.zip › TableS6 R2.pdf]

Table S6: dichotomous correlations for each immune marker.

variable 1	variable 2	test	normalization	p-value
Number of affected epitopes	GZMB	Wilcoxon Rank-Sum Test	None	0.2590
Number of affected epitopes	LCA	Wilcoxon Rank-Sum Test	None	0.5835
Number of affected epitopes	CD8	Wilcoxon Rank-Sum Test	None	0.1065
Number of affected epitopes	GZMB normalized	Wilcoxon Rank-Sum Test	To the total number of leucocytes (LCA)	0.7471
Number of affected epitopes	CD8 normalized	Wilcoxon Rank-Sum Test	To the total number of leucocytes (LCA)	0.6341
Number of affected epitopes	PD-L1 TPS	Wilcoxon Rank-Sum Test	None	0.4679
Number of affected epitopes	PD-L1 CPS	Wilcoxon Rank-Sum Test	None	0.6369
Number of affected epitopes	PD-L1 IC-Score	Wilcoxon Rank-Sum Test	None	0.7639

Supplementary Table S6: dichotomous correlations for each immune marker.

GRZMB: granzyme B, LCA: leucocyte common antigen (CD45), PD-L1: Programmed death-ligand 1, TPS: Tumor proportion score, CPS: combined positive score, IC-Score: Immune cell score.
